# Goals of Care Conversation Education Program: Impact on Resident Documentation

**DOI:** 10.1093/geroni/igaf122.2598

**Published:** 2025-12-31

**Authors:** Kelly Hsu, M Isabel Friedman, Jessica Cohen, Ann Eichorn, Mark Tursi, Christian Nouryan, Edith Burns

**Affiliations:** Northwell Health, New Hyde Park, New York, United States; Northwell Health, New Hyde Park, New York, United States; Northwell Health, New Hyde Park, New York, United States; Northwell Health, New Hyde Park, New York, United States; Northwell Health, New Hyde Park, New York, United States; Northwell Health, Great Neck, New York, United States; Zucker School of Medicine Hofstra-Northwell, Manhasset, New York, United States

## Abstract

Eliciting patient-centered goals of care (GOC) leads to tailored treatment plans, better outcomes, greater patient/family/provider satisfaction. Most educational approaches elicit immediate feedback on self-perceived change in knowledge and skills (Kirkpatrick level 1 and 2). Our health system implemented Goals of Care Conversation Education Program (GoCCEP) and wished to assess if GoCCEP impacted provider behavior (Kirkpatrick level 3). GoCCEP-trained resident physicians from 7 different residency programs (2 family med {FM}, 3 internal {IM}, 1 PMR, 1 surgery) compared to matched controls from same program/year of training who didn’t take the course. Total number of unique patients seen by each resident with at least 1 GOC note identified (numerator) over total number unique patients seen by that resident (denominator) 6 months pre-GoCCEP and 6 months post-GoCCEP. Inpatients included were aged ≥65 or LACE ≥12. Z scores for differences calculated with 2 proportions test. Total of 2,067 patients with GOC notes located for 76 GoCCEP and 301 Control residents (total 312,895 unique patients). GoCCEP-trained residents increased documentation pre-post 0.6% to 1.1% (p < 0.001). GoCCEP vs. control PGY-1: 1.3% vs. 0.6% (z=-10.17, p < 0.001); PGY-2: 1.1 vs. 0.7% (z=-2.96, p < 0.004); PGY 3: 0.9% vs. 0.6% (z=-3.78, p < 0.001); PGY-3 Chief/higher: 1.1 vs.0.23% (z=-8.1, p < 0.001). Trained FM residents had significantly higher documentation than IM residents, 3.23% vs. 0.45% (z = 13.00, p < 0.001). GoCCEP associated with increased GOC documentation though overall level of documentation low regardless of year of experience. Multiple factors likely underlie these observations suggesting need for multipronged education/interventions to increase patient-centered GOCC.

